# Supplementary material for: Detection of SARS-CoV-2–Specific Antibodies in Human Breast Milk and Their Neutralizing Capacity after COVID-19 Vaccination: A Systematic Review
Source: Int J Mol Sci. 2023 Feb 3;24(3):2957. doi: 10.3390/ijms24032957 (PMC9917673; doi:10.3390/ijms24032957)
Supplement: Supplementary file 1 [file ijms-24-02957-s001.zip › ijms-2176992-supplementary.pdf]

Supplementary Table S1. Local and systemic effects after anti-SARS-CoV-2 vaccination in lactating individuals

	**[1]**		**[2]**	**[3]**		**[4]**		**[5]**	**[6]**	**[7]**		**[8]**	**[9]**		**[10]**		**[11]**
**Dose**	1	2	-	1	2	1	2	-	-	1	2	-	1	2	1	2	-
***N* (lactating individuals)**	30		35	28		48		14	110	32		16	84		31		42
**Aches/Muscle pain**	17	27	43	18	14	21	69	57	N/A	19	59	N/A	N/A	N/A	13	52	21
**Adenopathy**	N/A	N/A	N/A	N/A	N/A	N/A	N/A	N/A	18	0	19	N/A	N/A	N/A	N/A	N/A	N/A
**General Asthenia**	N/A	N/A	N/A	N/A	N/A	N/A	N/A	N/A	N/A	N/A	N/A	N/A	N/A	N/A	N/A	N/A	29
**Injection site reaction/rash**	N/A	N/A	N/A	N/A	N/A	88	88	N/A	N/A	75	78	N/A	N/A	N/A	65	58	26
**Headache**	13	30	3	14	18	21	67	43	9	31	56	N/A	N/A	N/A	29	36	10
**Joint pain**	N/A	N/A	3	21	21	8	31	7	N/A	16	31	N/A	N/A	N/A	N/A	N/A	5
**Fever/Chills**	13	27	11	4	18	13	63	7	6	9/7	25/45	44	0	12	3	39	21/7
**Local pain**	N/A	N/A	N/A	21	21	N/A	N/A	36	2	N/A	N/A	N/A	48	41	N/A	N/A	N/A
**Nausea**	N/A	N/A	N/A	N/A	N/A	4	13	14	1	3	13	N/A	N/A	N/A	N/A	N/A	N/A
**Mastitis**	N/A	N/A	3	N/A	N/A	2	0	N/A	N/A	N/A	N/A	N/A	N/A	N/A	N/A	N/A	N/A
**Abdominal pain**	N/A	N/A	N/A	N/A	N/A	2	0	N/A	N/A	N/A	N/A	N/A	N/A	N/A	N/A	N/A	N/A
**Diarrhea**	N/A	N/A	N/A	N/A	N/A	4	4	N/A	N/A	N/A	N/A	N/A	N/A	N/A	N/A	N/A	N/A
**Tiredness**	13	37	N/A	7	25	44	81	0	N/A	28	53	N/A	10	33	13	45	N/A
**Sore throat**	0	7	N/A	N/A	N/A	N/A	N/A	N/A	N/A	N/A	N/A	N/A	N/A	N/A	N/A	N/A	N/A
**Rhinorrhea/Cough**	0	3	9	N/A	N/A	N/A	N/A	N/A	N/A	N/A	N/A	N/A	N/A	N/A	N/A	N/A	N/A
**Other**	N/A	N/A	N/A	7	32	N/A	N/A	7	11	N/A	N/A	N/A	14	26	0	23	N/A
**No side effects**	60	33	29	7		N/A	N/A	N/A	35	N/A	N/A	N/A	N/A	N/A	N/A	N/A	N/A
**No questionnaire answer**	13	10	0	25		N/A	N/A	N/A	N/A	N/A	N/A	N/A	N/A	N/A	N/A	N/A	N/A

References

1. Narayanaswamy, V.; Pentecost, B.T.; Schoen, C.N.; Alfandari, D.; Schneider, S.S.; Baker, R.; Arcaro, K.F. Neutralizing antibodies and cytokines in breast milk after coronavirus disease 2019 (COVID-19) mRNA vaccination. *Obstet. Gynecol.* **2022**, *139*, 181-191, doi:10.1097/AOG.0000000000004661.
2. Yeo, K.T.; Chia, W.N.; Tan, C.W.; Ong, C.; Yeo, J.G.; Zhang, J.; Poh, S.L.; Lim, A.J.M.; Sim, K.H.Z.; Sutamam, N.; et al. Neutralizing activity and SARS-CoV-2 vaccine mRNA persistence in serum and breastmilk after BNT162b2 vaccination in lactating women. *Front. Immunol.* **2021**, *12*, 783975, doi:10.3389/fimmu.2021.783975.
3. Juncker, H.G.; Mulleners, S.J.; van Gils, M.J.; Bijl, T.P.L.; de Groot, C.J.M.; Pajkrt, D.; Korosi, A.; van Goudoever, J.B.; van Keulen, B.J. Comparison of SARS-CoV-2-specific antibodies in human milk after mRNA-based COVID-19 vaccination and infection. *Vaccines (Basel)* **2021**, *9*, 1475, doi:10.3390/vaccines9121475.
4. Golan, Y.; Prahl, M.; Cassidy, A.G.; Gay, C.; Wu, A.H.B.; Jigmeddagva, U.; Lin, C.Y.; Gonzalez, V.J.; Basilio, E.; Chidboy, M.A.; et al. COVID-19 mRNA vaccination in lactation: Assessment of adverse events and vaccine related antibodies in mother-infant dyads. *Front. Immunol.* **2021**, *12*, 777103, doi:10.3389/fimmu.2021.777103.
5. Charepe, N.; Goncalves, J.; Juliano, A.M.; Lopes, D.G.; Canhao, H.; Soares, H.; Serrano, E.F. COVID-19 mRNA vaccine and antibody response in lactating women: A prospective cohort study. *BMC Pregnancy Childbirth* **2021**, *21*, 632, doi:10.1186/s12884-021-04051-6.
6. Lechosa-Muñiz, C.; Paz-Zulueta, M.; Mendez-Legaza, J.M.; Irure-Ventura, J.; Cuesta González, R.; Calvo Montes, J.; López-Hoyos, M.; Llorca, J.; Cabero-Pérez, M.J. Induction of SARS-CoV-2-specific IgG and IgA in serum and milk with different SARS-CoV-2 vaccines in breastfeeding women: A cross-sectional study in northern Spain. *Int. J. Environ. Res. Public Health* **2021**, *18*, doi:10.3390/ijerph18168831.
7. Jakuszko, K.; Kościelska-Kasprzak, K.; Żabińska, M.; Bartoszek, D.; Poznański, P.; Rukasz, D.; Kłak, R.; Królak-Olejnik, B.; Krajewska, M. Immune response to vaccination against COVID-19 in breastfeeding health workers. *Vaccines (Basel)* **2021**, *9*, 663, doi:10.3390/vaccines9060663.
8. Collier, A.Y.; McMahan, K.; Yu, J.; Tostanoski, L.H.; Aguayo, R.; Ansel, J.; Chandrashekar, A.; Patel, S.; Apraku Bondzie, E.; Sellers, D.; et al. Immunogenicity of COVID-19 mRNA vaccines in pregnant and lactating women. *JAMA* **2021**, *325*, 2370-2380, doi:10.1001/jama.2021.7563.
9. Perl, S.H.; Uzan-Yulzari, A.; Klainer, H.; Asiskovich, L.; Youngster, M.; Rinott, E.; Youngster, I. SARS-CoV-2-specific antibodies in breast milk after COVID-19 vaccination of breastfeeding women. *JAMA* **2021**, *325*, 2013-2014, doi:10.1001/jama.2021.5782.
10. Gray, K.J.; Bordt, E.A.; Atyeo, C.; Deriso, E.; Akinwunmi, B.; Young, N.; Baez, A.M.; Shook, L.L.; Cvrk, D.; James, K.; et al. Coronavirus disease 2019 vaccine response in pregnant and lactating women: a cohort study. *Am. J. Obstet. Gynecol.* **2021**, *225*, 303.e301-303.e317, doi:10.1016/j.ajog.2021.03.023.
11. Scrimin, F.; Campisciano, G.; Comar, M.; Ragazzon, C.; Davanzo, R.; Quadrifoglio, M.; Giangreco, M.; Stabile, G.; Ricci, G. IgG and IgA antibodies post SARS-CoV-2 vaccine in the breast milk and sera of breastfeeding women. *Vaccines (Basel)* **2022**, *10*, 125, doi:10.3390/vaccines10010125.
